# miR-638 suppresses DNA damage repair by targeting SMC1A expression in terminally differentiated cells

**DOI:** 10.18632/aging.100998

**Published:** 2016-07-12

**Authors:** Mingyang He, Yi Lin, Yunlan Tang, Yi Liu, Weiwei Zhou, Chuang Li, Guihong Sun, Mingxiong Guo

**Affiliations:** ^1^ College of Life Sciences, Wuhan University, 430072 Wuhan, P. R. China; ^2^ School of Basic Medical Sciences, Wuhan University, 430071 Wuhan, P.R. China

**Keywords:** miR-638, SMC1A, cell differentiation, DNA damage repair, drug sensitivity

## Abstract

The reduction of DNA damage repair capacity in terminally differentiated cells may be involved in sensitivity to cancer chemotherapy drugs; however, the underlying molecular mechanism is still not fully understood. Herein, we evaluated the role of miR-638 in the regulation of DNA damage repair in terminally differentiated cells. Our results show that miR-638 expression was up-regulated during cellular terminal differentiation and involved in mediating DNA damage repair processes. Results from a luciferase reporting experiment show that structural maintenance of chromosomes (SMC)1A was a potential target of miR-638; this was verified by western blot assays during cell differentiation and DNA damage induction. Overexpression of miR-638 enhanced the sensitivity of cancer cells to cisplatin, thus reducing cell viability in response to chemotherapy drug treatment. Furthermore, miR-638 overexpression affected DNA damage repair processes by interfering with the recruitment of the DNA damage repair-related protein, γH2AX, to DNA break sites. These findings indicate that miR-638 might act as a sensitizer in cancer chemotherapy and accompany chemotherapy drugs to enhance chemotherapeutic efficacy and to improve the chance of recovery from cancer.

## INTRODUCTION

Cells regularly encounter various DNA-damaging factors that give rise to genomic DNA damage throughout the life cycle of an organism. Cells systematically address both endogenous and exogenous sources of DNA damage through their conserved DNA repair and cell cycle checkpoint pathways, which allow cells to maintain genomic stability or prevent cells from entering mitosis. For cellular physiological and pathological processes, it is vital to maintain genomic integrity and DNA accuracy. To protect cell genomic integrity, different strategies are employed between in terminally differentiated cells or post-mitosis differentiating cells and in proliferating cells [1, 2]. In the face of stress, proliferating cells preferentially select apoptosis over DNA damage [1, 3]. However, in terminally differentiated cells, the transcription-coupled repair and differentiation-associated repair systems are retained, because these cells do not require genomic replication [1, 4]. Moreover, long-lived terminally differentiated astrocytes retain the DNA repair capacity of non-homologous end-joining [5]. Terminally differentiated muscle cells have not only been shown to exhibit decreased base excision repair capacity, which leads to the accumulation of DNA single-strand breaks [6], but are also resistant to ionizing radiation (IR) [7]. Short-lived terminally differentiated blood cells have reduced DNA repair abilities, due to the miR-24-mediated down-regulation of H2AX, which is a key DNA repair protein [8]. These studies indicated that depending on the distinct intercellular micro-environment, diverse terminally differentiated or post-mitosis differentiating cells have reduced DNA repair capacities and may employ different DNA damage repair pathways to deal with both endogenous and exogenous sources of DNA damage. However, the underlying molecular mechanisms behind the reduction of DNA repair capacity in terminally differentiated cells are poorly understood.

MicroRNAs (miRNAs) are a class of small (∼22 nt), noncoding RNAs that play important regulatory roles at the posttranscriptional gene regulation level via translational inhibition and mRNA destabilization [9, 10]. miR-638 is a primate-specific miRNA [11] that plays important roles in development, hematopoiesis, tumorigenesis, and leukemogenesis. Recently, miR-638 was proposed to play potential roles in embryonic development [12], human vascular smooth muscle cell proliferation and migration [13], hematopoietic differentiation [14], and lens development [15]. It may even affect tumorigenesis by targeting specific genes [16, 17]. The expression of miR-638 is involved in the development and progression of multiple types of tumors, such as gastric cancer [18-21], colorectal carcinoma [22, 23], non-small cell lung cancer [24, 25], basal cell carcinoma [26], nasopharyngeal carcinoma [27], and melanoma [28]. MiR-638 was also found to be involved in virus entry, replication, and propagation, and may serve as an antiviral molecule [29-35]. Moreover, miR-638 is stably expressed in human plasmas and may be a potential novel biomarker for leukemia and systemic lupus erythematosus [36-38]. Another study demonstrated that miR-638 is significantly up-regulated in three growth arrest states (premature senescence, replicative senescence, quiescence), particularly in replicative senescence, which suggests that miR-638 may repress pathways that control cell cycle progression and DNA repair [39]. Indeed, recent studies have implicated miR-638 in the response to radiotherapy and chemotherapy treatments [40-42], thereby demonstrating a role for miR-638 in the regulation of genes involved in DNA damage repair [43, 44]. Although these studies have demonstrated that miR-638 plays crucial roles in various physiological and pathological processes, the underlying molecular mechanisms remain unclear.

The structural maintenance of chromosomes (SMC) protein 1A (SMC1A, also known as SMC1), which is one of six SMC family members (SMC1–6), is a well-characterized gene that is linked to the proper cohesion and correct segregation of sister chromatids during cell division [45]. Inhibiting SMC1A expression efficiently inhibits the proliferation and colony formation of U251 and U87MG cells, and SMC1A silencing leads to S cell cycle arrest [46]. Knocking down SMC1A expression in U251 cells with SMC1A-targeted interfering RNAs inhibits cell growth and induces G2/M cell cycle arrest [47]. Recent studies found that cohesin SMC1/3 is also involved in the cellular DNA damage response pathway. SMC1A, together with the other cohesins SMC3, 5, and 6, physically tether the sister chromatids together, thus stabilizing strand invasion and facilitating homologous recombination-mediated repair [48]. Furthermore, Kitagawa et al. demonstrated that the ATM-BRCA1/NBS1-SMC1 pathway is critical for mediating cell survival and chromosomal stability after DNA damage [49].

In our previous work, we demonstrated that miR-638 regulates leukemic cell proliferation and differentiation by targeting cyclin-dependent kinase 2 (CDK2) expression. Furthermore, miR-638 may cause G1 cell cycle arrest [14]. The aim of this study was to evaluate the role of miR-638 in the crosslink processes of terminal differentiation and DNA damage repair, with regard to SMC1A expression.

## RESULTS

### miR-638 is up-regulated in terminally differentiated cells

**Figure 1:**
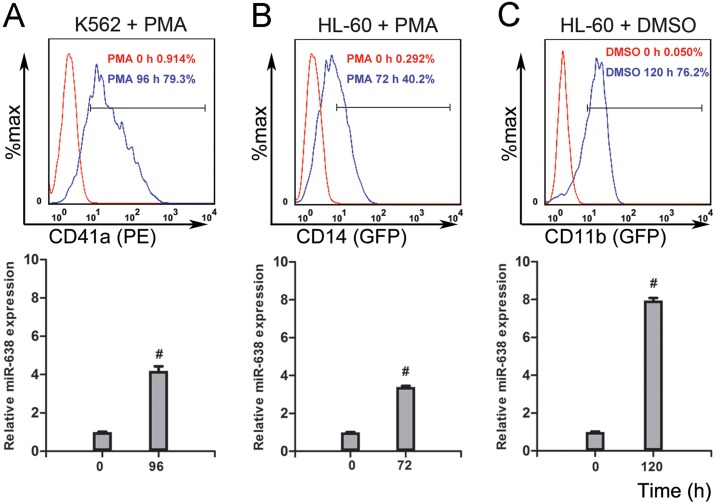
The expression of miR-638 is up-regulated in terminally differentiated cells **(A)** K562 cells were treated with or without phorbol myristate acetate (PMA) for 96 h, after which they were stained with a fluorescence-conjugated antibody against CD41a. The expression of CD41a was detected by flow cytometry. Levels of miR-638 in treated and untreated cells were detected by qRT-PCR. **(B)** HL-60 cells were treated with or without PMA for 72 h. Cells were then stained with a fluorescence-conjugated antibody against CD14, and the expression of CD14 was detected by flow cytometry. Levels of miR-638 in treated and untreated cells were detected by qRT-PCR. **(C)** HL-60 cells were treated with or without dimethyl sulfoxide (DMSO) for 120 h. Cells were then stained with a fluorescence-conjugated antibody against CD11b, and the expression of CD11b was detected by flow cytometry. Levels of miR-638 in treated and untreated cells were detected by qRT-PCR. The numbers of positively stained cells after PMA or DMSO induction are shown in the graphs. Comparative qRT-PCR was run in three duplicates per group, and the results were normalized to Snord44 snRNA. #: *p*<0.0001, compared to control with the Student's *t* test. *Error bars*, S.D.

In our previous study, we identified miR-638 as a novel regulator of myeloid differentiation in leukemic cells and presented its impacts on cell cycle arrest and proliferation [14]. Given that the differentiated cell is commonly regarded as having progressively attenuated DNA repair [2], and miR-638 is involved in DNA damage repair processes by targeting BRCA1 expression [16, 41], we intended to further explore the role of miR-638 in the DNA damage repair of terminally differentiated cells. We first measured the expression level of miR-638 in terminally differentiated myeloid cell lines. K562 is a typical acute myeloid leukemia cell line that can be induced by phorbol myristate acetate (PMA) to differentiate into mega-karyocytic-like cells. As shown in Figure 1A, along with the increased expression of the megakaryocyte-specific surface marker (CD41a) [50], the expression of miR-638 exhibited a four-fold increase at 96 hours after PMA-induced megakaryocytic differentiation when compared with that of untreated control cells. A noticeable up-regulation of miR-638 expression was also observed in another promyelocytic leukemic cell line, HL-60, which can differentiate into monocytic/macrophage- or granulocytic-like phenotypes by PMA or dimethyl sulfoxide (DMSO) treatment, respectively (Figure 1B and 1C). Increased macrophage- (CD14) or granulocytic-specific (CD11b) surface markers were measured by flow cytometry to confirm differentiation [51]. These results show that miR-638 expression was up-regulated during the terminal differentiation of cells.

### SMC1A and BRCA1 are two target genes of miR-638 and are down-regulated in terminally differentiated cells

**Figure 2:**
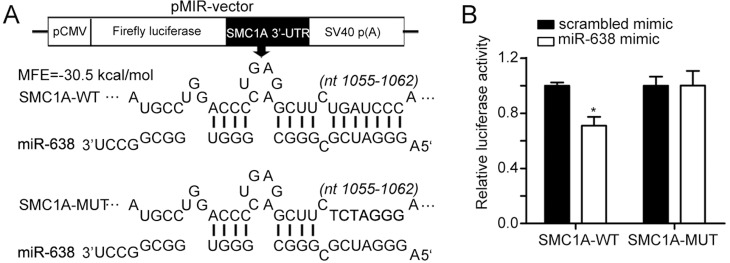
SMC1A is a target of miR-638 **(A)** The scheme illustrates the vector structure that was used for target validation and the predicted miR-638 binding sites in the 3′UTR of SMC1A mRNA. SMC1A-MUT has a seven-base mutation at the 3′UTR. **(B)** Luciferase activities were measured and normalized by Renilla luciferase expression. Data are presented as relative luciferase activity. *: *p*<0.05, compared to control with the Student's *t* test. *Error bars*, S.D.

To investigate the functional mechanism of miR-638 in cellular differentiation, we performed TargetScan analysis [52]. We selected SMC1A, which is a member of the structural maintenance of chromosomes family [53], as a potential target gene of miR-638. Using RNAhybrid [54], a putative binding site in the 3′-UTR of SMC1A was predicted to have a minimum free energy hybridization of −30.5 kcal/mol (Figure 2A). To verify this prediction, a luciferase reporting experiment was conducted. We constructed two luciferase reporter vectors that harbored the wild-type 3′-UTR of SMC1A (SMC1A-WT) or the mutant 3′-UTR of SMC1A (SMC1A-MUT) (Figure 2A). In comparison with the control (scrambled mimic), transient transfection with miR-638 mimics caused a 30% reduction in luciferase activity for the wild-type 3′-UTR of SMC1A (SMC1A-WT). However, the mutation in the 3′-UTR had almost no inhibitory effect on the luciferase activity of overexpressed miR-638 (Figure 2B). Therefore, we concluded that miR-638 could directly regulate SMC1A by targeting its 3′-UTR region.

**Figure 3:**
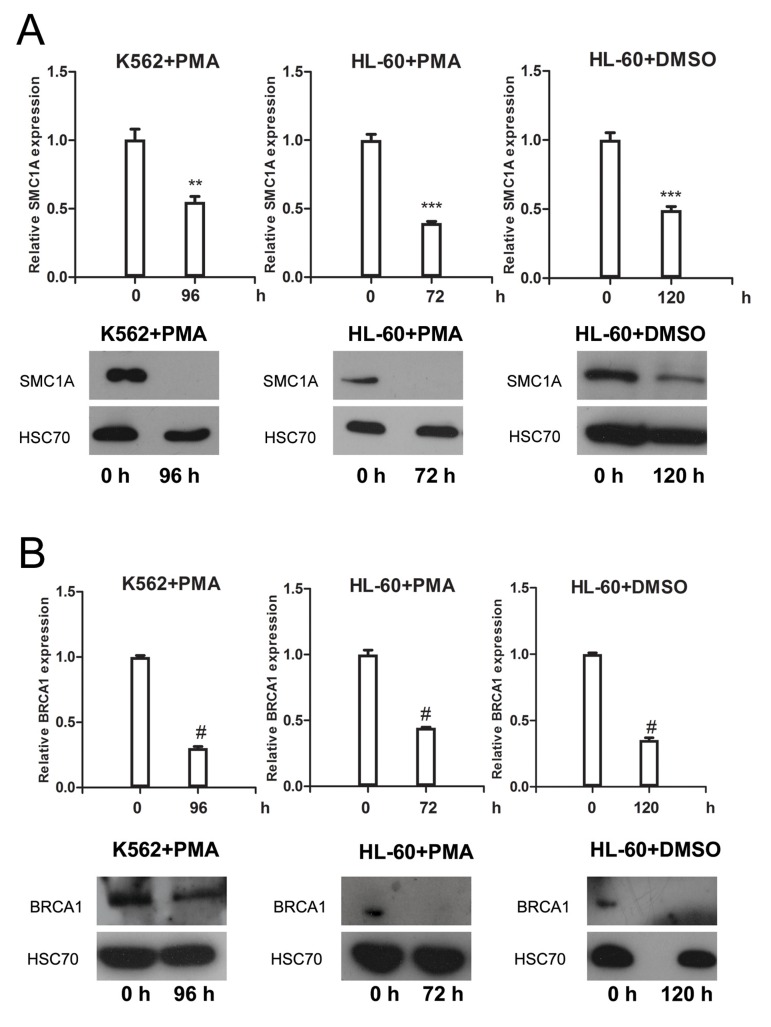
SMC1A and BRC1A expression levels are down-regulated in terminally differentiat-ed cells **(A)** The mRNA and protein levels of SMC1A were measured by qRT-PCR and western blotting, respectively, in PMA-treated K562 cells, PMA-treated HL-60 cells, and DMSO-treated HL-60 cells. **(B)** The mRNA and protein levels of BRCA1 were measured by qRT-PCR and western blotting, respectively, in PMA-treated K562 cells, PMA-treated HL-60 cells, and DMSO-treated HL-60 cells. Each group of comparative qRT-PCR contained three duplicates, and the results were normalized to GAPDH mRNA. HSC70 served as the loading control. **: *p*<0.01, ***: *p*<0.001, #: *p*<0.0001, compared to control with the Student's *t* test. *Error bars*, S.D.

To further verify SMC1A as a novel target gene of miR-638, we measured the expression change of SMC1A in terminally differentiated myeloid cells. The mRNA and protein expression levels of SMC1A were significantly decreased in K562 and HL-60 cells after PMA- or DMSO-induced differentiation (Figure 3A). We also tested the expression level of BRCA1, which is another miR-638 target gene [43], in differentiated myeloid cells. Similarly, induced differentiation noticeably decreased BRCA1 mRNA and protein expression (Figure 3B).

All of these findings confirmed that the expression of miR-638 was inversely correlated with the expression of SMC1A, thus suggesting that SMC1A and BRCA1 are the target genes of miR-638 during cell differentiation.

### miR-638 overexpression attenuates DNA repair ability in terminally differentiated cells

**Figure 4:**
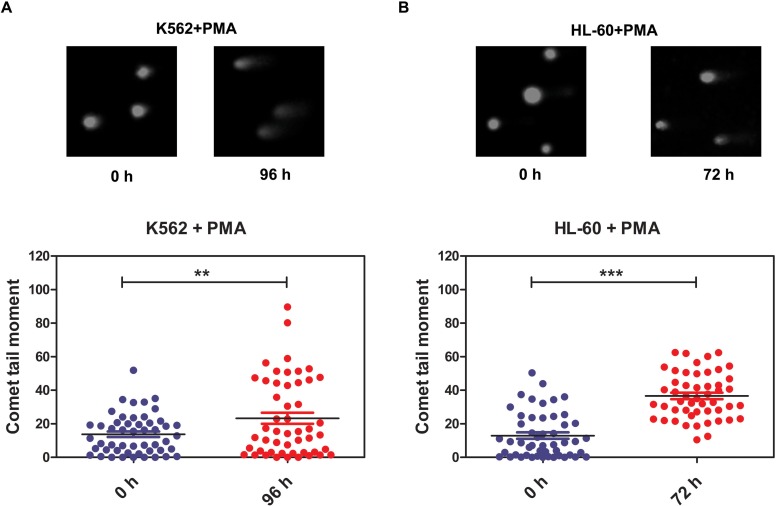
DNA damage repair ability is reduced in terminally differentiated cells **(A)** DNA damage was measured by the comet assay in PMA-treated K562 cells. **(B)** DNA damage was measured by the comet assay in PMA-treated HL-60 cells. Representative blots are shown. The comet tail moment was counted, and 50 cells were analyzed in each group. ***: *p*<0.001, compared to control with the Student's *t* test. *Error bars*, S.D.

**Figure 5:**
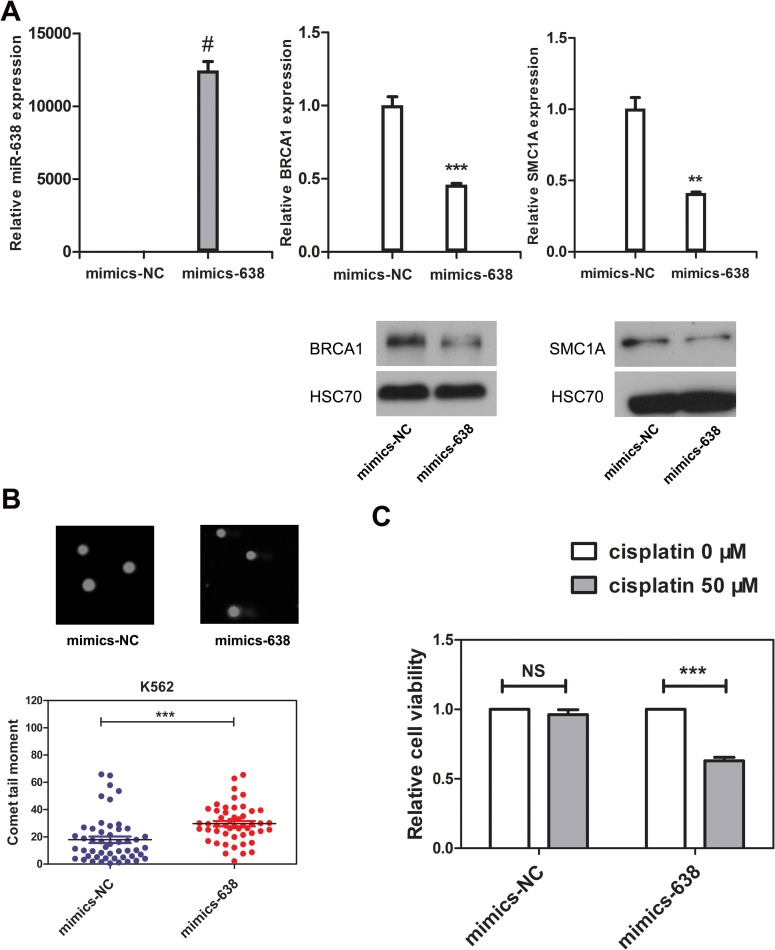
The overexpression of miR-638 in K562 cells impedes DNA damage repair ability and increases the cellular sensitivity to cisplatin **(A)** K562 cells were transfected with negative control mimics (mimics-NC) or miR-638 mimics (mimics-638). Levels of miR-638 in the transfected cells were measured by qRT-PCR, and the levels of SMC1A and BRCA1 mRNA and protein were detected by qRT-PCR and western blotting, respectively. Each group of comparative qRT-PCR contained three duplicates, and the results were normalized to Snord44 snRNA. HSC70 served as the loading control. **(B)** K562 cells were transfected with mimics-NC or mimics-638, after which they were treated with cisplatin (5 μM) for 18 h. DNA damage was then measured by the comet assay. Representative blots are shown. The comet tail moment was counted, and 50 cells were analyzed in each group. **(C)** K562 cells were transfected with mimics-NC or mimics-638 and then re-plated in 96-well plates. Afterwards, they were treated with cisplatin (0 or 50 μM), which was dissolved in RPMI-1640 medium, for 48 h, and the relative cell viability was measured by the CCK8 assay. Data are presented as a column chart. NS: not significant, **: *p*<0.01, ***: *p*<0.001, #: *p*<0.0001, compared to control with the Student's *t* test. *Error bars*, S.D.

The DNA repair process was recently reported to be dependent on cell type and differentiation stage. In terminally differentiated cells, DNA damage repair is less efficient. However, the mechanisms by which DNA repair is regulated during differentiation are still unclear [2, 55]. Here, we show that miR-638 expression was significantly up-regulated in differentiated cells. Previously, the studies showed that miR-638 affects DNA repair by mediating BRCA1 expression [16, 41]. We then hypothesized that miR-638 might play a role in impeding DNA repair ability during cell differentiation. The persistence of DSBs is an indicator of unrepaired DNA damage, and we performed the comet assay in differentiated K562 and HL-60 cells to measured this. The comet tail moment quantifies the extent of unrepaired DNA damage [8]. Consistent with the previously reported study [56], we found an significant increase in the comet tail moment of terminally differentiated cells following cisplatin treatment (Figure 4A and 4B). Next, we examined the comet tail moment of K562 cells that were transfected with transient miR-638 mimics. The transfection efficiency was evaluated by qRT-PCR. The expression of miR-638 was apparently increased in mimics-638-transfected cells, and the mRNA and protein levels of its targeting genes (SMC1A and BRCA1) were accordingly down-regulated (Figure 5A). At 48 hours after transfection, cells were treated with cisplatin to induce DNA damage, and the comet tail moment was measured after 18 h. As shown in Figure 5B, compared to cells transfected with control mimics (mimics-NC), a significant increase in comet tail moment is detected after cisplatin treatment.

Because of the impairment in DNA repair ability, the sensitivity of the cell to genotoxic drugs will increase. Next, we tested the cell viability of miR-638-overexpressing K562 cells. Cisplatin (50 μM) treatments had almost no effect on the viability of mimics-NC-transfected cells (Figure 5C). However, miR-638 overexpression noticeably down-regulated cell viability. We attributed this down-regulation of cell viability to the inhibitory effect of miR-638 overexpression on DNA repair.

**Figure 6:**
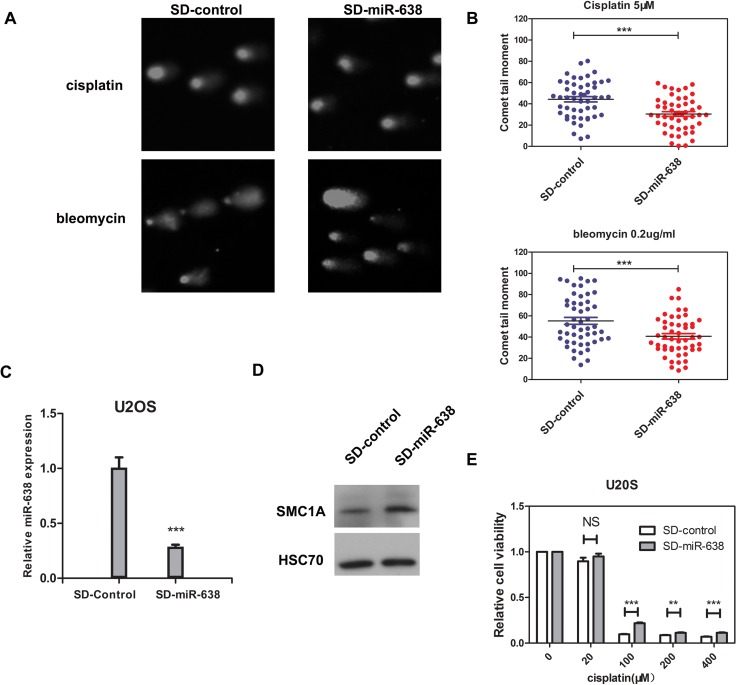
The down-regulated miR-638 in U2OS cells enhances DNA damage repair ability and reduces the cellular sensitivity to cisplatin **(A)** U2OS cells that transfected with control virus (SD-control) or retroviruses expressing miR-638 (SD-miR-638) were treated with cisplatin (5 μM) or bleomycin (0.2 μg/ml) for 18 h, and DNA damage was measured by comet assay after 18 h. Representative blots are shown. **(B)** The comet tail moment of the cells in (A) were analyzed. The comet tail moment was counted, and 50 cells were analyzed in each group. **(C)** The expression levels of miR-638 in the virus transfected cells were measured by qRT-PCR. **(D)** The protein levels of SMC1A in the virus transfected cells were measured by western blotting. **(E)** SD-control or SD-miR-638 U2OS cells were re-plated on 96-well plates and treated with five final concentrations of cisplatin (0, 20, 100, 200, or 400 μM), which were dissolved in DMEM medium, for 48 h. Relative cell viability was measured by the MTT assay. Data are presented as a column chart. NS: not significant, **: *p*<0.01, ***: *p*<0.001, compared to control with the Student's *t* test. *Error bars*, S.D.

In addition, we performed the comet assay and cell viability measurement in U2OS cell, which is an ideal model for studying DNA damage repair processes. Because U2OS cells express high levels of endogenous miR-638 [17], we generated a stable U2OS cell line by retrovirus transfection, in which the level of miR-638 was inhibited for about 70% (Figure 6C). The protein level of SMC1A was then accordingly increased, thus confirming the suppressed miR-638 expression (Figure 6D). Contrary to miR-638-overexpressing K562 cells, the down-regulation of miR-638 in U2OS cells evidently decreased the comet tail moment following bleomycin or cisplatin treatments (Figure 6A and 6B). Moreover, the cellular sensitivity to a high con-centration of cisplatin (≥100 μM) was reduced in miR-638-down-regulated cells (SD-miR-638), although a low concentration of cisplatin (20 μM) had no effect on cell viability (Figure 6E).

Overall, these results demonstrated a modulatory effect of miR-638 on DNA repair ability during cell differentiation.

### miR-638 affects DNA repair ability and cell sensitivity to cisplatin by mediating SMC1A expression

**Figure 7:**
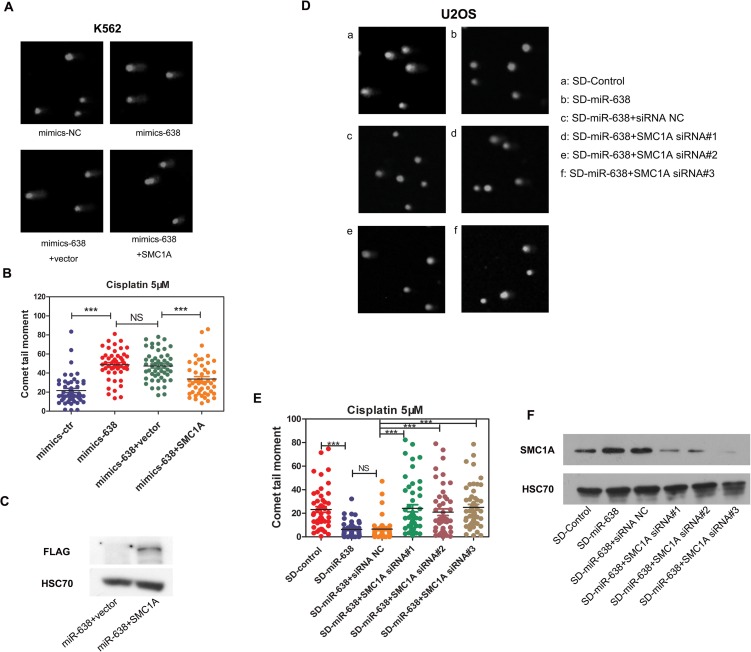
The miR-638-mediated suppression of SMC1A affects DNA damage repair ability **(A)** K562 cells were transfected with mimics-NC or mimics-638. Mimics-638-transfected cells were then co-transfected with SMC1A plasmid or vector plasmid. Cells were treated with cisplatin (5 μM) for 18 h, and the DNA damage in collected cells was analyzed by the single-cell comet assay. Representative blots are shown. **(B)** The comet tail moment was counted, and 50 cells were analyzed in each group of **(A)**. **(C)**The plasmid used in rescuing SMC1A expression has a 3×Flag tag, and the efficiency of rescue was measured by assessing Flag protein levels via western blotting. **(D)** SD-control or SD-miR-638 U2OS cells were transfected with SMC1A siRNA (#1, #2, #3) or negative siRNA. After 48 h transfection cells were treated with cisplatin (5 μM) for 18 h, and the DNA damage in collected cells was analyzed by the single-cell comet assay. Representative blots are shown. **(E)** The comet tail moment was counted, and 50 cells were analyzed in each group of (D). **(F)** The efficiency of the siRNA transfection in U2OS cells was measured via western blotting. NS: not significant, ***: *p*<0.001, compared to control with the Student's *t* test. *Error bars*, S.D.

Kitagawa et al. previously confirmed the role of SMC1A in the ATM-NBS1-BRCA1 pathway in response to DNA damage after IR [49]. SMC1A, which forms a complex with BRCA1, can be recruited at DNA break sites and then phosphorylated by ATM [49]. Therefore, to test whether the effect of miR-638 on DNA repair is partly regulated via the reduction in SMC1A expression, we restored SMC1A protein levels in miR-638-overexpressing K562 cells by co-transfecting cells with an exogenous plasmid that carried the coding sequence (CDS) region of SMC1A cDNA (without the miR-638 binding region, 3′-UTR). In addition, three Flag tags were constructed at the 3′ end of the SMC1A CDS region to allow for the detection of transfection efficiency using a Flag antibody. A noticeable Flag band of the exogenous SMC1A plasmid-transfected group indicated that the suppressed SMC1A protein level, which was caused by increased miR-638 expression, was restored (Figure 7C). We found that the comet tail moment in miR-638-overexpressing cells was higher than that in negative control-transfected cells, and co-transfection with the vector plasmid had no effect on tail moments. However, when miR-638-overexpressing cells were co-transfected with the SMC1A plasmid, a 40% reduction in the comet tail moment was significantly observed (Figure 7A and 7B), which indicated the decreased of unrepaired DNA damage.

We further verified this on miR-638 down-regulated U2OS cells (SD-miR-638). The increased protein level of SMC1A in SD-miR-638 U2OS cells has already been proved (Figure 6D), and siRNAs against SMC1A were transfected into SD-miR-638 U2OS cells. The knockdown efficiency of siRNAs was validated by western blot, and the significant knockdown of SMC1A protein expressions were detected (Figure 7F). We found that the knockdown of SMC1A (by siRNA#1, #2, #3) increased the comet tail moment of SD-miR-638 U2OS cells, whose unrepaired DNA damage was reduced owing to miR-638 down-regulation (Figure 7D and 7E).

These findings suggest that the obstruction of miR-638 on DNA repair ability is partly mediated by its effect on SMC1A protein expression.

### miR-638 expression influences γH2AX expression and foci formation in the nucleus

**Figure 8:**
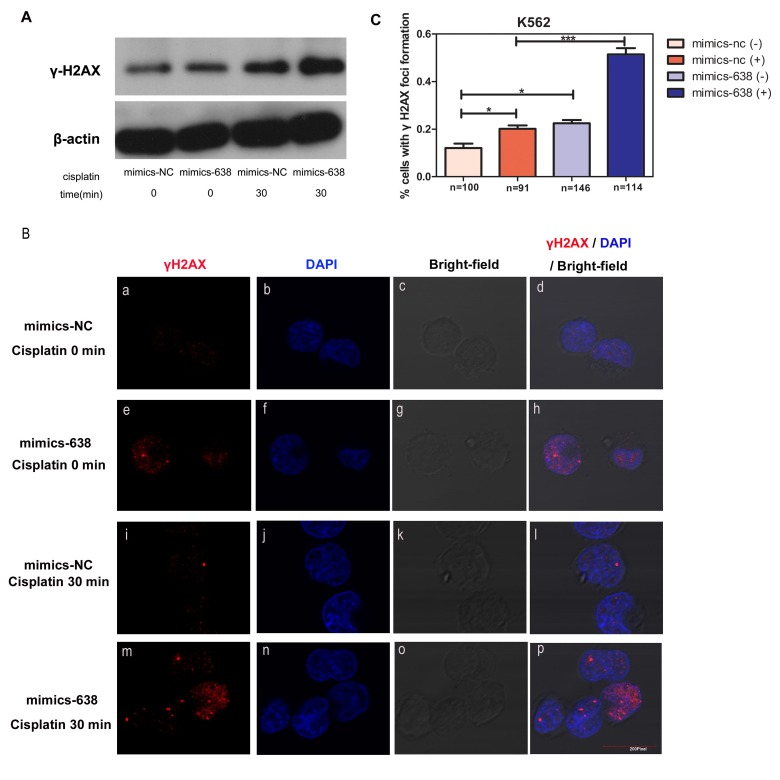
The overexpression of miR-638 affects γH2AX protein expression and foci formation **(A)** K562 cells were transfected with mimics-NC or mimics-638 and then exposed to cisplatin (25 μM) for 30 min. The protein level of γH2AX was assessed via western blotting. β-actin served as the loading control. **(B)** The same treated K562 cells in **(A)** were collected to analyze γH2AX (red) localization in the cell nucleus by immunofluorescence microscopy. The nucleus was visualized by DAPI (blue) staining. **(C)** The number of cells with γH2AX foci formation was counted, and the statistical number of each group was shown below (n). NS: not significant, *: *p*< 0.05, ***: *p*<0.001, compared to control with the Student's *t* test. *Error bars*, S.D.

H2AX has been identified as one of the key histones to undergo various post-translational modifications in response to DNA double-strand breaks (DSBs) and its phosphorylation and recruitment at DNA break sites serves as an indicator of DNA damage repair processes [57, 58]. Thus, we detected the protein level and nuclear foci formation of γH2AX to identify the mechanism by which miR-638 mediates DNA damage repair processes. First, we observed an increase in γH2AX protein levels in mimics-638-transfected cells (Figure 8A). Simultaneously, an enhanced γH2AX foci formation in the nucleus was observed in mimics-638-transfected cells (Figure 8B a-h, 8C), suggesting that miR-638 may play a role in promoting γH2AX protein expression and foci formation in the nucleus. In addition, we tested the protein level and nuclear foci formation of γH2AX in both mimics-638- and mimics-NC-transfected cells following 30 min of cisplatin treatment. Upon transient cisplatin treatment, the protein levels of γH2AX in both mimics-638- and mimics-NC-transfected cells were increased. Moreover, this up-regulation in mimics-638-transfected cells was even more evident than that in control cells (Figure 8A), indicating that miR-638 can promote the cellular DNA damage response to DSBs. Consistently, the foci formations of γH2AX in the nucleus were also augmented after 30 min of cisplatin treatment, and the overexpression of miR-638 made this recruitment even more obvious (Figure 8B i-p, 8C).

These findings confirmed the influence of over-expressed miR-638 on DNA damage repair and showed that miR-638 affected the protein expression and recruitment of the DNA repair-related protein, γH2AX.

## DISCUSSION

In the previous study, miR-638 was identified to play a role in regulating target genes (e.g., PLD1, CDK2, p53, PTEN, BRCA1, SOX2, Sp2, TSPAN1) to regulate various cellular processes, including cellular proliferation, cell cycle arrest, apoptosis, differentiation, DNA repair, and tumorigenesis [14, 16, 17, 20, 21, 23, 24, 28, 59]. In this study, we further verified the participation of miR-638 in the DNA damage repair process of terminally differentiated cells and its positive regulation of the cellular sensitivity to cisplatin.

Differentiated cells do not exhibit genomic DNA replication, and most of their genes are silent. These cells have been suggested to maintain genetic integrity by gathering enough cellular energy from the removal of DNA damage from the non-essential bulk of their genome [2]. Therefore, clarifying the variation of the DNA repair process in differentiated cells may allow us to better understand the relationships among genomic integrity, differentiation, and senescence, and may even provide new theoretical supports in regenerative medicine [60]. Changes in the expression levels of miRNAs can rapidly and effectively regulate many cellular activities, including the altered DNA repair process in differentiated cells. These expression changes differ between dividing cells and differentiated cells. miR-183, miR-96, and miR-182 are significantly up-regulated in terminally differentiated myeloid cells [61]. During myoblast-to-myocyte differentiation, miR-214 is down-regulated, whereas miR-135 is over-expressed [62]. Our results showed that the unrepaired DNA damage in terminally differentiated myeloid cells were more serious than undifferentiated cells and the expression level of miR-638 was up-regulated, suggesting that the expression change of miR-638 in terminally differentiated cells is closely associated with the increased unrepaired DNA damage. Indeed, the overexpression of ectogenic miR-638 mimics in K562 cells significantly increased unrepaired DNA damage and cellular sensitivity to cisplatin, which indicated a negative correlation between the expression of miR-638 and cellular DNA repair ability in terminally differentiated cells. Moreover, it will be worthwhile to determine whether the negative regulation of miR-638 on DNA repair ability exists in all cell types or only in terminally differentiated cells. Interestingly, exogenous down-regulation of miR-638 in U2OS cells similarly impeded unrepaired DNA damage and cellular sensitivity to cisplatin.

In this present study, we identified SMC1A as a novel target gene of miR-638. SMC1A is a downstream effector in the ATM-NBS1-BRCA1 pathway that regulates the G1/S phase checkpoint. ATM and ATR can phosphorylate SMC1A, which depends on the phosphorylation of BRCA1 and NBS1 [45, 63]. The phosphorylation of SMC1A has been reported to lead to reduced chromosomal breakage and increased cell survival [49], thus suggesting a role for SMC1A in DNA damage repair. Indeed, the negative regulation of miR-638 on DNA repair ability was blocked by the restoration of SMC1A in both K562 and U2OS cell. Furthermore, we observed that only about 4 fold increase of miR-638 level is correlated with non-detectable protein levels of SMC1A in Figure 3A. However, the exogenous 1000 fold-increased miR-638 in K562 cells caused a mild protein level decrease (Figure 5A). Generally, these results suggest that there may be other mechanisms not only miR-638 contributes to the changes of SMC1A.

Other effectors in the ATM-NBS1-BRCA1 pathway were also detected. Up-regulation of miR-638 caused an inconspicuous change of the ATM protein level (data not shown), whereas the significant overexpression and enhanced foci formation of γH2AX were detected between miR-638-overexpressing and normal groups. As a major adaptor protein in DNA damage response, γH2AX takes part in DNA damage response processes by facilitating the accumulation and retention of repair factors and chromatin modifying factors at the site of damage [58]. In addition, the mediation of other target genes, such as BRCA1 and p53, by miR-638 has been shown to affect tumorigenesis and the DNA damage response [16, 17]. These findings suggest that miR-638 plays an active role in DNA damage response and DNA repair process, but how miR-638 regulates this process by affecting the interrelation between target genes and signal transmission at the unitary level is a further issue that we are interested in exploring.

It is well known that chemotherapy is a general and significant therapy for cancer treatment. Unfortunately, some types of carcinoma are initially insensitive and become resistant to chemotherapy drugs, thus weakening the therapeutic efficacy. It is becoming increasingly important to discover assistant drugs or therapeutic methods that can enhance chemotherapeutic efficacy. Based on a greater propensity to accumulate DNA damage and the genomic instability of tumors, DNA damage-related chemotherapy, such as carbo-platin and cisplatin, has historically been exploited in cancer treatment [64, 65]. Because miRNAs are important endogenous gene modulators in regulating the DNA damage response, they have potential applications to be used as sensitizers in cancer chemotherapy [66-68]. miR-302b has been reported to enhance breast cancer cell sensitivity to cisplatin by regulating ATM, which is an important serine/threonine kinase that transduces DNA damage signals to downstream effectors in the DNA damage response [69]. In the present study, the overexpression of miR-638 promoted the protein expression and foci formation of the early DNA damage response effector, γH2AX, at DNA break sites. As a result, the cellular DNA damage response was enhanced. Consistently, the cellular sensitivity to cisplatin was positively correlated with the expression of miR-638 in both K562 and U2OS cells. These findings indicate that miR-638 might act as a sensitizer in cancer chemotherapy and accompany chemotherapy drugs to enhance chemotherapeutic efficacy and to improve the chance of recovery from cancer.

## MATERIALS AND METHODS

### Cell culture and differentiation

K562 cells and HL-60 cells were grown in RPMI-1640 supplemented with 10% (v/v) fetal bovine serum (FBS). U2OS cells were grown in Dulbecco's Modified Eagle Medium (DMEM) supplemented with 10% (v/v) FBS. We treated K562 cells (5×10^5^ cells/ml) with PMA (10 mg/ml, 96 h) for differentiation into megakaryocytes. HL-60 cells (5×10^5^ cells/ml) were treated with PMA (120 nM, 72 h) or DMSO (1.25%, 120 h) for differentiation into macrophages or granulocytes, respectively.

### RNA isolation and quantitative real-time RT-PCR

Total RNA was obtained using the Direct-zol RNA Miniprep (ZYMO RESEARCH, USA), and 1 μg total RNA was subjected to reverse transcription using the FastQuant RT Kit (with gDNase) (TIANGEN, China). Quantitative analyses of mRNA levels were performed using SYBR Select Master Mix (Life Technology, USA). Primers are provided in Table 1. Reverse transcription and quantitative analyses of miR-638 were performed using the one-step miRNA cDNA Synthesis Core Reagent Kit and SYBR Premix HS SM-Taq (Geneup, Shenzhen, China). GAPDH and Snord44 small nuclear RNA were used as internal controls for mRNA and miRNA quantification, respectively. The levels of mRNAs and miRNAs were determined by the 2^−ΔΔCt^ method.

**Table 1:** Primers used in this study

Name	Sequences (5′ to 3′)
Primers used in qRT-PCR	
GAPDH-FP	TCAACGACCACTTTGTCAAGCTCA
GAPDH-RP	GCTGGTGGTCCAGGGGTCTTACT
BRCA1-FP	CCCTCAAGGAACCAGGGATG
BRCA1-RP	GCTGCACGCTTCTCAGTGGT
SMC1A-FP	ATGCTGCCTTGGATAACA
SMC1A-RP	ATCACACAGTCCCCTTGC
Primers used in plasmid construction	
SMC1A-CDS-FP	TTTGGATCCCCGCCATGGATGGGGTTCCTGAAACTGAT
SMC1A-CDS-RP	TTTCTCGAGCTGCTCATTGGGGTTGGG
638-sites-FP	CGCGTAGGGATCGCGGGCGGGTGGCGGCCTAGGGATCGCGGGCGGGTGGCGGCCTA
638-sites-RP	AGCTTAGGCCGCCACCCGCCCGCTAGGCCGCCACCCGCCCGCGATCCCTA
SMC1A-MUT-FP	CTTCTGTCCCTAATAGCTCCCTAGAGAAGCTCTCAGGGGTCC
SMC1A-MUT-RP	GGACCCCTGAGAGCTTCTCTAGGGAGCTATTAGGGACAGAAG

### Oligonucleotide transfection

Negative control mimics (mimics-NC) and miR-638 mimics (mimics-638) were designed and synthesized (Geneup, Shenzhen, China). Mimics were transfected into K562 cells at working concentrations of 100 nM using Lipofectamine 3000 (Invitrogen, USA). A fragment from the CDS of SMC1A mRNA (without the 3′-UTR region), which disrupted the binding sites by miR-638, was cloned into the pCNV-3 tag-8 expression vector (Stratagene, USA). Mimics-638 (100 nM) and the SMC1A plasmid (2 μg) or vector plasmid (2 μg) were co-transfected into K562 cells using Amaxa nucleofection (Amaxa, Germany). SMC1A siRNA (#1 CGGCGTATTGATGAAATCAAT, #2 CCAACATTGATGAGATCTATA, #3 TAGGAGGTTCTTCTGAGTACA) or negative control siRNA were transfected into U2OS cells using Lipofectamine 3000 (Invitrogen, USA).

### Generation of stably transfected U2OS cells

Retrovirus designs and packaging were performed by Geneup (Shenzhen, China). U2OS cells (80%) were seeded on 6-well plates. After U2OS cells are at 80% confluency, packaged retroviruses were added into fresh DMEM medium and cultured with U2OS cells for 24 h. and then fresh DMEM medium was added in retroviruses transfected cells. Puro-resistant gene was cloned into the retroviral vector and successfully transfected stable cell lines were selected by puromycin treatment.

### Luciferase reporter assay

A fragment from the 3′-UTR of SMC1A mRNA harboring putative miR-638 binding sites was cloned into multiple sites of firefly luciferase (pMIR-REPORT luciferase vector) (Applied Biosystems, USA). To test whether SMC1A mRNA is directly regulated by miR-638, the mutant version with a seven-base mutation via overlapping PCR was also cloned into the vector. HEK293T cells were seeded on 24-well plates (1×10^5^ cells/well) and transfected with 100 ng of the reporter vectors, 10 ng pRL-TK, and miR-638 or negative control mimics (final concentration: 100 nM) using Lipofectamine 2000 reagent (Invitrogen, USA). After 48 h, we detected luciferase activities using the Dual Luciferase Assay System (Promega, USA).

### Cellular viability assay

After 48 hours transfection of mimics-NC or mimics-638, K562 cells (6,000 cells per well) were re-plated on 96-well plates and treated with one of two final concentrations of cisplatin (0 or 50 μM) for 48 h. Cisplatin was dissolved in RPMI-1640. The CCK8 assay kit reagent (Promoter Biological, China) was added into every well (10 μl per well) and incubated for 2 h at 37°C, after which the optical density was read at 450 nm with a microplate reader (Biotek, USA). Each group had five replicates. Stably transfected U2OS cells (6,000 cells per well) were seeded on 96-well plates and separately treated with five concentrations of cisplatin (0, 20, 100, 200, or 400 μM) for 48 hours. Cisplatin was dissolved in DMEM. MTT ([3-4,5-dimethyl-2-thiazolyl]-2,5-diphenyl-2-H-tetra-zolium bromide; 10 μl of a 5 mg/ml solution) was added into every well and incubated for 4 hours at 37°C, and then the generated crystals were dissolved by DMSO. The optical density was read at 450 nm with a microplate reader (Biotek, USA), and each group had five replicates.

### Comet assay

We induced double-strand breaks by treating cells with cisplatin (5 μM) or bleomycin (0.2 μg/ml) for 18 h at 37°C. Treated cells were collected and washed twice with phosphate-buffered saline (PBS). We performed the single-cell comet assay, according to the manufacturer's instructions (KeyGEN BioTECH, China). First, 0.5% normal-melt agarose was spread on frosted glass slides. After the agarose solidified, we spread 0.7% low-melt agarose with 10^5^ cells/ml on the first gelatin. While waiting for the second agarose coating to solidify, we spread the 0.7% low-melt agarose on the second agarose and solidified the gelatin at 4°C for 30 min. After the agarose solidified, the slides were successively placed in Lysis Buffer containing 10% DMSO at 4°C for 1 h. The slides were washed with PBS three times and placed in EDTA NaOH electrophoresis liquid at room temperature for 40 min. Electrophoresis was run for 20 min at 25 V. Slides were then washed with 0.4 mol/L Tris-HCl buffer solution three 10-min intervals at 4°C. Cells were stained with propidium iodide for 10 min in the dark. Nuclei were visualized using a fluorescence microscope and analyzed with CometScore. Fifty cells were counted in each experiment.

### Protein extraction and immunoblotting

Proteins were extracted from cell lines using RIPA buffer, which was supplemented with protease inhibitors. Protein extracts were fractionated by SDS-PAGE or Tricine-SDS-PAGE using 8% polyacrylamide gels and transferred to polyvinylidene fluoride membranes (Millipore, USA). The membranes were blocked with 5% skim milk in TBS with Tween 20 (1:2000, V/V) (TBST) for 1 h at room temperature. The membranes were then incubated with primary antibodies at 4°C overnight. Afterwards, the membranes were washed with TBST three times and then incubated with secondary antibodies for 1 h at room temperature. After washing five to six times, membranes were incubated with electrochemi-luminescence western blotting substrate (Bio-red) and visualized in the darkroom. The following primary antibodies were used: anti-phosphorylated-Histone H2AX (Ser139) antibody (JBW301; Upstate; 1:2,000), anti-SMC1 (8E6) Mouse monoclonal antibody (#6892, Cell Signaling, 1:1,000), and anti-BRCA1 antibody (#9010, Cell Signaling, 1:1,000).

### Immunofluorescence microscopy

K562 cells were transfected with mimics and then treated with cisplatin (25 μM) for 30 min. Afterwards, cells were collected and washed with PBS. Cells were then suspended in the wash buffer (10^5^ cells/μl) and spread on the cover glass. After the cells adhered on the glass, the glass slides were fixed with 4% paraformaldehyde in PBS for 15 min at room temperature, permeated with 0.2% Triton X-100 in PBS for 15 min at room temperature, and then blocked with 5% bovine serum albumin in PBS for 30 min at room temperature. Cells were incubated with primary antibodies at 4°C overnight. Afterwards, the cells were washed with PBS three times and incubated with secondary antibodies (Cy3 goat anti-mouse IgG) for 30 min in the dark. Cells were then stained with DAPI (1 μg/ml) in PBS for 3 min at room temperature. The anti-phosphorylated-Histone H2AX (Ser139) antibody (JBW301; Upstate, 1:1,000) was used.

### Statistical analysis

Data are presented as means ± standard deviation of triplicates or duplicates. Differences between two groups were analyzed by the Student's *t* test (unpaired, two tails), and a p<0.05 value was considered as significant. All data analyses were performed with GraphPad Prism version 5.0 (GraphPad Software, USA).
